# Demoralization, depression and anxiety in postpartum women of culturally and linguistic diverse backgrounds in Australia

**DOI:** 10.18332/ejm/140791

**Published:** 2021-10-07

**Authors:** Adeel Arshad, Katia Foresti, Matheus M. Rech, Vlasios Brakoulias, Carlos Zubaran

**Affiliations:** 1 Department of Mental Health, Western Sydney Local Health District, Sydney, Australia; 2 Sunnyside Clinic, Sydney, Australia; 3 Hornsby Specialist Centre, Sydney, Australia; 4 School of Medicine, University of Caxias do Sul, Caxias do Sul, Brazil; 5 School of Medicine, Western Sydney University, Sydney, Australia; 6 School of Medicine, The University of Notre Dame, Sydney, Australia

**Keywords:** postpartum period, puerperal disorders, anxiety, depression, cultural diversity

## Abstract

**INTRODUCTION:**

The present study aims to investigate whether mothers from Culturally and Linguistically Diverse (CALD) backgrounds present with higher levels of demoralization in comparison with their non-minority counterparts, and to explore potential correlations between demoralization and anxiety as well as depression in the same sample of mothers.

**METHODS:**

Women admitted to a public tertiary care teaching hospital were invited to participate in the study within 24–48 hours following delivery. The study compared women who did not regard English as their main spoken language to native English-speaking women. Women were asked to complete the demographic Kissane Demoralization Scale (KDS) and Being a Mother Scale (BaM-13) questionnaires. Participants were contacted by phone, 6 to 8 weeks after they had completed the KDS and the BaM-13 questionnaires, to complete an Edinburgh Postnatal Depression Scale (EPDS) and State Trait Anxiety Scale (STAI) questionnaires.

**RESULTS:**

Mothers of CALD background presented with significantly higher scores on the KDS (p<0.001), STAI (p<0.001) and EPDS (p<0.001) scales in comparison with their non-CALD counterparts. Furthermore, when mothers were reassessed after 6 to 8 weeks, higher KDS scores in the postnatal period predicted significantly higher anxiety and depression scores, according to STAI (p<0.001) and the EPDS (p<0.001), respectively.

**CONCLUSIONS:**

The results of this study reveal that, mothers of CALD background manifest higher levels of demoralization as well as anxiety and depression in the postpartum period when compared with their non-CALD counterparts.

## INTRODUCTION

Mothers in the postpartum period are known to experience a series of hormonal, physical and psychological changes. Alterations of mood and emotions have long been recognized to be highly prevalent in the postpartum period^1^. These alterations in mood and emotions have also been associated with potentially detrimental consequences to mothers’ dyadic bonding with their babies and child’s development^2^.

Australia is one of the world’s most culturally diverse modern democracies. Since 1945, over 7.5 million people have migrated to and settled in Australia. In 2015, the resident Australian population born overseas was estimated to be 28.2% of the total population^3^. Immigrants become domiciled in Australia via official programs for skilled workers and their families as well as the humanitarian program for refugees and those in refugee-like circumstances.

The frequently stressful and at times traumatic events that many migrant women may have faced during their relocation journeys, in conjunction with acculturation tensions experienced during their adaptation processes, may predispose women from culturally and linguistically diverse (CALD) backgrounds to endure prominent levels of anxiety and depression in the postpartum period^4^. Factors such as minority status, limited proficiency in the predominant local language, and limited social support may expose migrant women to an increased risk for perinatal mental disorders^5^. Results from a population-based survey of primiparous women in Australia, in which immigrant women whose primary language was not English were also investigated, revealed that demoralization was associated with adverse postnatal experiences and some of these associations remained significant even after demographics and other distress related measures were controlled for^4^. The results of the latter study were interpreted as further evidence of the satisfactory discriminant validity of the construct of demoralization in research with postpartum women^6^. In the respective study, the authors also aimed at investigating demoralization in order to obtain a comprehensive understanding of women’s experiences in the postpartum period beyond the changes in symptoms of anxiety and depression^6^.

Mothers in the postpartum period may also experience demoralization, which is a psychological state affecting individuals under stress, which leads to feelings of helplessness and hopelessness^7^. The significant physical and psychological changes that women experience in the postpartum period may further exacerbate feelings of social isolation and hopelessness, particularly among immigrants. Evidence from studies in developed^8^ and developing countries^9^ have confirmed that immigrant mothers are at an increased risk for developing mental health disorders due to the additional challenges and burdens associated with minority and immigrant status.

In this study, the aim was to investigate the prevalence of demoralization in women of CALD background during the postpartum period by conducting a comparative analysis with mothers of non-CALD background. Potential correlations between demoralization and depression and/ or anxiety were also explored. The hypothesis was that mothers of CALD background would present with higher levels of demoralization, anxiety and depression in the postpartum period and that there would be a significant correlation between demoralization and depression and/ or anxiety. It has been demonstrated that women who feel particularly demoralized perceived the assessment of demoralization as relevant to their circumstances, given that such assessment can provide meaningful information which is not usually captured in the evaluation process of depression and anxiety^6^. In light of the significant obstacles imposed on women who had to migrate and adapt to living circumstances in other countries, the assessment of demoralization in this study is particularly pertinent and relevant.

## METHODS

### Objectives

The main objective of this study was to investigate whether mothers from a CALD background presented with higher levels of demoralization in comparison with their non-minority counterparts. An additional objective in this study was to explore potential correlations between demoralization and anxiety as well as depression in the same sample of mothers.

### Study design and sample

Participants were surveyed in the maternity service at Nepean Hospital, a large tertiary referral hospital located in Penrith, New South Wales, Australia. The sample included 75 women who were born in a country where English is not the primary language and a consecutively recruited group of 75 women who were born in Australia or spoke English as their first language. Inclusion criteria required mothers to be giving birth to a healthy baby in singleton pregnancies. Exclusion criteria included an unstable medical condition; being deemed too mentally unstable to participate in this study and lacking competence to give consent. This study was approved by the Nepean Hospital Human Research Ethics Committee and conducted under the oversight of the Research Governance Officer. Participants gave written informed consent to participate.

### Research instruments

#### Demographic Survey Form (DSF)

Demographic data collected in this study included information about the participants’ age, marital or relationship status, place of birth, first language, household annual income, as well as delivery method and breastfeeding status.

#### The Kissane Demoralization Scale (KDS)

The KDS is a 24-item, 5-point response self-report questionnaire. There is concurrent validity between this demoralization scale and measures of existential distress, hopelessness and depression. The KDS scale was scored as per the guidelines of Kissane et al.^10^. Higher KDS scores indicate higher level of demoralization. A cut-off score ≥24 was used to classify the mothers as being demoralized.

#### The Being a Mother Scale (BaM-13)

The BaM-13 is a 13-item self-report scale that measures women’s experience of early motherhood. It helps identify the women’s experience of her child, her experience of herself as an adult and her emotional closeness with her child. The BaM-13 was scored as per the guidelines of Matthey^11^. Higher BaM scores indicate higher level of stress. A cut-off score >9 is used to classify the experiences of the mother as stressful.

#### The State-Trait Anxiety Inventory (STAI)

The STAI is a brief 6-item scale used to assess state anxiety. The STAI shows significant criterion, discriminant, and predictive validity in perinatal populations. The STAI was scored as per the guidelines by Julian^12^. Higher STAI scores indicate higher level of anxiety. A cut-off score ≥55 was used to classify the mothers as being anxious.

#### The Edinburgh Postnatal Depression Scale (EPDS)

The EPDS is a 10-item self-report instrument that requires mothers to rate their mood in the preceding seven days. The Edinburgh Postnatal Depression Scale was scored as per the guidelines proposed by Cox et al.^13^. Higher scores indicate phenomena compatible with depression. A score of 0–9 was classified as absence of postnatal depression, 10–12 as borderline postnatal depression, and a score of ≥13 was classified as postnatal depression.

All instruments have been validated in the English language and used to assess several different populations of mothers, including women in the postpartum period; the cut-off scores of all research tools were kept unaltered.

### Data collection

Research workers conducted reviews of demographic information of women admitted to the postpartum ward, within 24–48 hours following delivery (Table 1), with emphasis on country of origin and language spoken at home. Newly admitted CALD as well as matching non-CALD women were verbally invited to participate in the study. Women were then informed about the study characteristics and research procedures; consent was obtained from those willing to participate in the study. The assistance of an interpreter was provided over the phone to women with limited command of spoken English (n=3). Participants were guaranteed privacy during their participation in the interviews. In the initial interview (Table 1) CALD and non-CALD women self-completed the questionnaires in the presence of research workers. When an interpreter was required, instruments were administered verbally. In the second interview (Table 2) questionnaires were administered over the phone. Upon consent, participants were asked to complete the DSF, as well as the KDS and the BaM-13 questionnaires. Research instruments were not translated to other languages. After 6–8 weeks from the initial interview (Table 2), participants were contacted by phone and requested to complete the EPDS and the STAI. Out of the 150 mothers, 7 mothers (3 CALD and 4 non-CALD) were not contactable. As a result, an additional 7 mothers (3 CALD and 4 non-CALD) were randomly recruited into the study and contacted after 6–8 weeks following the initial interview. Out of this second cohort, 1 mother from the CALD subgroup could not be contacted and another research participant was recruited. Participants were recruited to ensure a sample size of 146 calculated a priori in an allocation rate equal to 1 with the following parameters: effect size d=0.5, α error probability=0.03, and power=0.8. Additional women were recruited to ensure a sufficient sample in the event of missing subjects.

**Table 1:** Demographic aspects of CALD and non-CALD Women

*Characteristics*	*CALD (n=75)*		*non-CALD (n=75)*			
	*Mean (range)*	*SD*	*Mean (range)*	*SD*	*t*	*p*
**Age** (years)	31.51 (19–44)	5.43	29.00 (19–38)	4.94	-2.957	0.004
	***Median (range)***	***IQR***	***Median (range)***	***IQR***	***U***	***p***
**Gestational age** (weeks)	37 (27–41)	2	37 (31–41)	3	2432.5	0.0359
**Pregnancies** (n)	2 (1–5)	1	1 (1–6)	1	2359	0.061
**Births** (n)	1 (1–5)	1	2 (1–4)	2	2284.5	0.029
**Living children** (n)	1 (1–5)	1	2 (1–4)	1	2332	0.046
	***n (%)***		***n (%)***		***χ^2^***	***p***
**Previous diagnosis**						
Depression	3 (4)		4 (5.3)		0.15	0.699
Anxiety	5 (6.6)		2 (2.7)		1.35	0.246
Depression and anxiety	1 (1.3)		5 (6.7)		2.78	0.096
**Education level**					157.00	<0.001
Primary	3 (4)		0 (0)		-	-
Secondary	15 (20)		10 (13.3)		-	-
Tertiary	57 (76)		65 (86.7)		-	-
**Household annual income** (AUD)					92.82	<0.001
20000–39999	6 (8)		10 (13.3)		-	-
≥40000	69 (92)		65 (86.7)		-	-

SD: standard deviation. IQR: interquartile range. AUD: 100 Australian dollars about 74 US$. t: Student’s t-test; U: Mann-Whitney U test. χ^2^: chi-squared.

**Table 2:** Country of birth and language spoken of CALD women

*Country of birth*	*n (%)*
Afghanistan	1 (1.3)
China	1 (1.3)
Egypt	5 (6.7)
Fiji	2 (2.7)
Greece	12 (16)
India	1 (1.3)
Iran	1 (1.3)
Iraq	7 (9.3)
Lebanon	1 (1.3)
Liberia	2 (2.7)
Malta	1 (1.3)
Nepal	4 (5.3)
Pakistan	6 (8.0)
Philippines	9 (12)
Samoa	1 (1.3)
Sierra Leone	1 (1.3)
Sudan	4 (5.3)
Tonga	3 (4.0)
Turkey	1 (1.3)
Vietnam	1 (1.3)
Zimbabwe	1 (1.3)

**Table:** 

*Language spoken*	*n (%)*
***at home***	
Afrikaans	1 (1.3)
Arabic	10 (13.3)
Dari	2 (2.7)
Dinka	1 (1.3)
English	1 (1.3)
Tagalog	10 (13.3)
Greek	2 (2.7)
Hindi	17 (22.7)
Creole	1 (1.3)
Maltese	2 (2.7)
Mandarin	1 (1.3)
Maori	1 (1.3)
Samoan	12 (16)
Sudanese	3 (4.0)
Tamil	1 (1.3)
Tongan	3 (4.0)
Urdu	6 (8.0)
Vietnamese	1 (1.3)

### Statistical analysis

Statistical analyses were conducted using Statistical Package for the Social Sciences (SPSS, IBM), Version 24. Prior to conducting statistical analysis, the assumptions of parametric statistics were inspected for all continuous variables. The Shapiro-Wilk test was used to check the statistical significance of normal distribution of the continuous variables. When applicable, parametric and non-parametric statistics and techniques were utilized.

Demographic statistics were generated by using summary statistics or frequency counts by the two cohorts. The comparison of the KDS scores between the two samples was conducted using a Mann-Whitney U test. A multivariate analysis of covariance (MANCOVA), with categorical independent variables and multiple dependent variables, was used to test for any significant effect of subgroup (i.e. non-CALD and CALD mothers) on depression (as measured by the EPDS) and anxiety (as measured by the STAI score), while controlling for demoralization (as measured by the KDS score), stressful circumstances of being a mother (as measured by the BaM-13 score) and age as covariate.

Potential associations between demoralization and anxiety, and depression were explored by using multiple linear regressions. Two multiple linear regression models were used to evaluate the predictive power of variables evaluated in Table 1 (BaM-13 and KDS) with regard to outcomes in Table 2 (STAI or EPDS). Participant subgroup (CALD and non-CALD), demoralization, education level (tertiary or lower), previous diagnostic, age, BaM-13 and KDS scores were considered as input for independent variables in both the models. Independent or explanatory variables were selected for inclusion into the multiple linear regression models using a stepwise method and a 0.05 criterion of statistical significance. Analyses of effect size were conducted according to criteria proposed by Cohen^14^.

## RESULTS

### Demographic statistics

Table 1 presents the demographic characteristics in each subgroup of women as well as comparisons between the characteristics presented by the subgroups. In summary, women had similar age range in both subgroups; vaginal delivery was more frequent in non-CALD subgroup and the rate of complications during the delivery was more frequent among the CALD women. The majority of women in the non-CALD subgroup had just one child (n=45; 60%); the majority of women in both subgroups were married: 80% (n=60) and 61.3% (n=46) in the CALD and non-CALD subgroups, respectively. Regarding previous diagnosis to depression, anxiety or both, among the CALD women 4% (n=3) had the previous diagnosis of depression; 6.7% (n=5) and 1.3% (n=1) had been diagnosed with and an anxiety disorder or both disorders, respectively, totaling to 14.7% of the sample already diagnosed for one of the conditions presented in the study. Among the subjects of the CALD subgroup, previous diagnosis had rates of 5.3% (n=4) for depression, 2.7% (n=2) for anxiety and 6.7% (n=5) for both, totaling to 12% (n=9) of individuals previously diagnosed with any of respective conditions. The most common countries of birth of CALD mothers were India (n=12; 16%), the Philippines (n=9; 12%) and Iraq (n=7; 9.3%). General data about CALD women’s country of birth and language spoken at home are presented in Table 2.

### Sample distribution

Shapiro-Silk test revealed statistically significant results for BaM-13 (p<0.001), KDS (p<0.001), STAI (p<0.001), and EDPS (p<0.001) scores; age did not reach statistical significance (p=0.05).

### Parametric and non-parametric analyses

Student’s t-test was conducted to compare age values between the two subgroups, having yielded a statistical significance (t= -2.96, p<0.001). Mann-Whitney U tests were performed for each assessment tool, with the following results: BaM-13 (U=1965.00, p<0.001), KDS (U=1327.00, p<0.001), STAI (U=1457.00, p<0.001) and EDPS (U=1746.50, p<0.001).

### Assessment tools’ total scores

In summary, women in the CALD subgroup demonstrated higher median scores in all the research tools. The stratification of participants according to the cut-off scores of each assessment tool revealed the following results: according to BaM-13, 12% of CALD women (n=9) and 2.7% of non-CALD women (n=2) reported having had stressful experiences during motherhood, whereas as per KDS scores, none of CALD (n=0) and 2.7% of the non-CALD women (n=2) were classified as demoralized. Regarding STAI scores, 41.33% of CALD (n=31) and 10.66% of non-CALD women (n=8) presented with scores compatible with anxiety. Finally, with regard to EPDS scores, 5.33% of the CALD (n=4) and 4% of the non-CALD women (n=3) presented with scores compatible with postnatal depression (borderline). Assessment tools’ medians and respective interquartile ranges for each subgroup are presented in Table 3.

**Table 3:** Score medians and IQR in each sample subgroup

*Instrument*	*CALD women*		*Non-CALD women*	
	*Median (range)*	*IQR*	*Median (range)*	*IQR*
BaM-13	1 (0–15)	4	0 (0–17)	0
KDS	7 (0–22)	9	0 (0–26)	4
STAI	48 (39–91)	25	41 (0–73)	4
EPDS	0 (0–12)	6	0 (0–12)	1

IQR: interquartile range.

### Analysis of correlations

As shown in the Table 4, the correlation coefficients of all four assessment tools are higher than 0 in all correlations, showing a positive interrelation among all scales, with statistical significance. The majority of correlation coefficients were higher in the non-CALD subgroup compared to those of the CALD. Yet, the correlation coefficients between BaM-13 and KDS (r=0.679, p<0.001) and between BaM-13 and STAI (r=0.505, p<0.001) were higher in the CALD subgroup. The highest correlation coefficient in the non-CALD subgroup was found between STAI and EPDS scores (r=0.722, p<0.001).

**Table 4:** Correlations* among the scores of CALD and non-CALD women

*Scale*	*BaM-13*		*KDS*		*STAI*		*EPDS*	
	*p*	*r*	*p*	*r*	*p*	*r*	*p*	*r*
**CALD**								
BaM-13		1	<0.001	0.679	<0.001	0.505	0.034	0.245
KDS	<0.001	0.679		1	<0.001	0.508	0.005	0.323
STAI	<0.001	0.505	<0.001	0.508	<0.001	1	<0.001	0.605
EPDS	0.034	0.245	0.005	0.323	<0.001	0.605		1
**Non-CALD**								
BaM-13		1	<0.001	0.671	<0.001	0.489	<0.001	0.64
KDS	<0.001	0.671		1	<0.001	0.525	<0.001	0.683
STAI	<0.001	0.489	<0.001	0.525	<0.001	1	<0.001	0.722
EPDS	<0.001	0.64	<0.001	0.683	<0.001	0.722		1

* Spearman’s correlation coefficients.

### Multiple regression analyses

The skewness for STAI and EPDS was 1.28 and 1.40, respectively. Kurtosis for STAI and EPDS revealed values of 0.57 and 1.04, respectively. Therefore, normality (Kurtosis and Skewness ≤2) was assumed. The results of a linear regression, in which STAI scores were defined as a dependent variable, revealed a general fitness of 48% (R^2^-adjusted=0.488, p<0.001). In addition, BaM-13 (95% CI: 0.16–1.38, p=0.13) and KDS (95% CI: 0.37–1.11, p<0.001) scores as well as the subgroup (CALD or non-CALD) variable (95% CI: -2.19 – -8.53, p<0.001) yielded significant effects on STAI scores. Age (p=0.573), previous diagnosis (p=0.67) and education level (p=0.77) were not statistically significant according to this analysis.

The second regression analysis was performed to assess the impact of subgroup, anxiety and motherhood-related stress on depression scores during postnatal period (EPDS). The model described 33% (R^2^-adjusted=0.33, p<0.001) of the actual observations. The BaM-13 score (p=0.42), age (p=0.73), previous diagnosis (p=0.79) and education level (p=0.70) did not reach statistical significance. KDS scores (95% CI: 0.21–0.32, p<0.001) and subgroup category (95% CI: -0.01–1.91, p=0.54) revealed an independent association with depression in the postnatal period. Both models were considered strong according with Cohen’s criteria.

## DISCUSSION

To our knowledge, this is the first study in which demoralization was investigated among women of CALD and non-CALD characteristics in the postpartum period. The results revealed that mothers in the CALD subgroup presented with significantly higher levels of anxiety and depression, which is consistent with evidence obtained elsewhere, as described below. Results reported by two other research groups revealed high rates of postnatal depression among mothers of culturally diverse groups in comparison with mother of predominant ethnic groups^9,15^. Evidence obtained elsewhere also revealed that postnatal depression might affect up to 42% of immigrant mothers, in contrast with the lower rate of 15% observed among local women^15^. Similarly, results from a study conducted in Australia revealed that immigrant women reported poorer postnatal experiences when compared with their Australian-born counterparts^16^.

The results of the present study also revealed that the maternal experience was perceived as more stressful among women in the CALD subgroup. The reasons to explain such discrepancy are not entirely known. Mothers of culturally diverse characteristics may face a series of obstacles, either in the region where this study was conducted, and/ or possibly other regions in Australia. A qualitative study of 20 West African migrant women, living in Sydney, revealed that the challenges pertaining to the adaptation to the norms of the predominant culture may cause an adverse impact on the health status of women of minority status^17^. The detrimental impact of adverse personal experiences and unfair hindrances, such as racism and discrimination, may also produce an unfavorable effect on the perinatal health outcomes as well as on the child’s health status and development^18,19^. Therefore, it is possible that, along with the tensions related to the obstacles of acculturation into the new adopted country, mothers of minority characteristics may also present with demoralization, anxiety and depression in the postpartum period.

The correlation between demoralization and symptoms of anxiety and depression, as observed in this study, is consistent with previous findings^6^, where the application of a demoralization scale was considered useful by health practitioners to better understand the psychological dimensions of the postnatal experience. In the investigation reported here, the importance of measuring demoralization in postpartum period has also been demonstrated.

The results of a study conducted in Canada with 119 pregnant immigrant women revealed that 42% of the participants presented with symptoms suggestive of depression, which substantiates the notion that the challenges inherent to migration may pose a high risk of depression to pregnant women^20^. Low socioeconomic status, which often represents an additional burden on migrant women, has also been cited as a potential risk factor for postpartum depression^21^. The importance of understanding the sociocultural contexts of childbirth has been mentioned as an important step towards the development of strategies to address the psychological distress women may experience in the postpartum period^22^.

Many migrant women in Australia may experience financial insecurity as a result of the obstacles they face to secure suitable jobs. Some women also financially support family members troubled by financial stress in their homeland. Even though mothers in the CALD subgroup are more likely to suffer from demoralization, anxiety and postpartum depression, results from a study in Canada^23^ reveal that immigrant women of diverse ethnic groups do not seek help when suffering from symptoms of postpartum depression. The results of the same study also revealed that the health services did not always respond appropriately to the mental healthcare needs of migrant women in the postpartum period^23^.

Immigrant women may have a distinct set of personal needs, given the uniqueness of one’s cultural values and belief systems, with different expectations with regard to mental healthcare^22,21^. If health services remain ill-equipped to appropriately respond to the specific necessities of women of culturally diverse backgrounds, this cohort of healthcare consumers will probably experience dissatisfaction with the quality of healthcare provided, which may reinforce further feelings of demoralization. It has been observed that women of culturally diverse standings in Australian society are less likely to access help to deal with symptoms of anxiety and depression^24^. Cultural competence has been defined as the ‘ability of systems to provide care to patients with diverse values, beliefs and behaviors, including tailoring delivery to meet patients' social, cultural and linguistic needs’^25^. The concept of cultural safety instead focuses on themes related to a history of colonial oppression and alienation, which allows the perpetuation of health inequalities^26^. These conceptual developments must be integrated into policy-making and clinical practice in order to guarantee optimal quality of care for all individuals.

### Limitations

Potential limitations of this study include the fact that its design was neither conceptualized to identify the potential reasons associated with higher scores of demoralization, anxiety and postpartum depression, nor to investigate the key risk factors associated with these results. Also, the EPDS and the STAI were not administered at the initial interview along with all the other measures, which prevented us from examining whether earlier states of depression and anxiety would have significantly predicted depression and anxiety 6 to 8 weeks later. Furthermore, research instruments were not translated and adapted to other languages; most of the research tools utilized in this study have not been validated to all languages spoken by research participants in this study. Finally, research instruments that assess demoralization, such as the KDS, include items that are also characteristic of depression. An independent assessment of subjective incompetence, which refers to one’s perception of incapacity to express feelings that would be appropriate in a stressful situation^27^, would have helped to further differentiate depression from demoralization.

### Future perspectives

Even though the KDS had already been validated in Australia as a useful assessment tool for measuring demoralization among women in the postpartum period^28^, the results of the present study reveal that CALD women may be particularly affected by demoralization when compared with their non-CALD counterparts. This unique aspect of the current study points to the need for further investigations to be conducted so that a better understanding of the specific challenges faced by CALD women in the postpartum period is obtained. The results of the current study also indicate that policies must be developed and implemented in order to facilitate access of women from culturally and linguistic diverse communities to comprehensive mental healthcare.

## CONCLUSIONS

The results presented in this study lend support to the hypothesis that mothers of culturally and linguistically diverse groups, in the postpartum period, present with higher scores of demoralization as well as higher levels of anxiety than their non-CALD counterparts. These findings highlight the importance of understanding the particular nature of the maternal experiences of CALD women in the postpartum period. The development of additional research in this area is necessary and strongly recommended.

## Data Availability

The data supporting this research are available from the authors on reasonable request.
